# Saturated fatty acid regulated lncRNA dataset during *in vitro* human embryonic neurogenesis

**DOI:** 10.1016/j.dib.2018.10.101

**Published:** 2018-10-27

**Authors:** Mustafa T. Ardah, Shama Parween, Divya S. Varghese, Bright Starling Emerald, Suraiya A. Ansari

**Affiliations:** a Department of Biochemistry, College of Medicine and Health Sciences, UAE University, Al Ain, Abu Dhabi, UAE; b Department of Anatomy, College of Medicine and Health Sciences, UAE University, Al Ain, Abu Dhabi, UAE

## Abstract

Human embryonic stem cells (hESCs) were used as a model of embryonic neurogenesis to identify the effect of excess fat uptake on neurodevelopment (Ardah et al., 2018). Herein, by directed differentiation of hESCs into neurons using established protocols, this data was generated for expression profiles of select lncRNAs during *in vitro* embryonic neurogenesis and their differential expression due to excess fat (palmitate) uptake. The undifferentiated hESCs were treated with 250 µM palmitate after identifying it as the highest concentration which is non-toxic to these cells. The palmitate treated hESCs were differentiated towards neurons keeping the levels of palmitate consistent throughout the differentiation process and fat uptake was confirmed by Oil Red O staining. The expression analysis of lncRNAs was performed by RT-qPCR on vehicle control and palmitate treated cells from 4 stages of differentiation, D0 (undifferentiated hESCs), D12 (neural stem cells), D44 (neural progenitors) and D70 (neurons) using lncRNAs array plates from Arraystar Inc. which contains 372 functionally identified lncRNAs found to be associated with lipid metabolism and other pathways (Cat# AS-NR-004).

**Specifications table**

**Table:** 

Subject area	Biology
More specific subject area	Stem cells
Type of data	Table (.xlsx), image (microscopy along with text)
How data was acquired	EVOS XL Core microscope, TECAN Infinite M200 pro plate reader, QuantStudio 7 Flex real time PCR machine (Applied Biosystems)
Data format	Analyzed
Experimental factors	Cells were treated with palmitate and vehicle control (ethanol).
Experimental features	Undifferentiated hESCs were treated with palmitate (250 µM unless indicated otherwise) and differentiated into cortical neurons *in vitro* using established protocols [1], [2], [3]. The cells were collected from 4 stages (D0, D12, D44 and D70) of differentiation and total RNA was isolated. After cDNA synthesis, long-noncoding RNAs (lncRNAs) were amplified by RT-qPCR using Long non-coding RNAs (LncRNAs) array plates from Arraystar, Inc. - USA (Cat# AS-NR-004). The data are shown in excel sheet (Supplementary Table 1) as log2 fold changes in palmitate treated cells relative to vehicle control after normalizing with 18S rRNA.
Data source location	Al Ain, Abu Dhabi, UAE
Data accessibility	The data is with this article.
Related research article	Ardah, M.T., et al.*, Saturated fatty acid alters embryonic cortical neurogenesis through modulation of gene expression in neural stem cells. J Nutr Biochem., 62 (2018) 230–246*doi.org/10.1016/j.jnutbio.2018.09.006[1]

**Value of the data**

• This is the first data to show expression profile of several functionally identified lncRNAs in a human model of embryonic neurogenesis.
• The data was generated to find differential expression of particular lncRNAs due to treatment with excess fat (palmitate). Thus this data could be studied further to understand their effect on cellular metabolism or other biological processes during embryonic neurogenesis.
• Since gene targets and biological processes/disease association of many of these lncRNAs is known, this data can be used to study the mechanism of action of particular lncRNAs in pathology, especially metabolic diseases in other models of metabolic syndrome.

## 1 Data

**Fig. 1:**
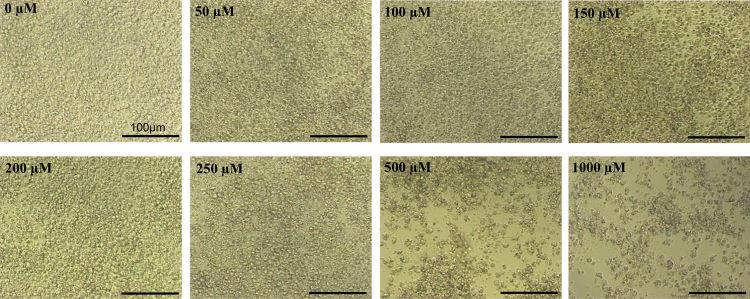
Effect of increasing palmitate concentrations on viability of hESCs. The H9 cells, grown on matrigel coated tissue culture dishes were treated with 0, 50, 100, 150, 200, 250, 500, 1000 μM of palmitate for 3 days. The phase images (20×) were captured on EVOS XL Core microscope (scale bar = 100 μm).

**Fig. 2:**
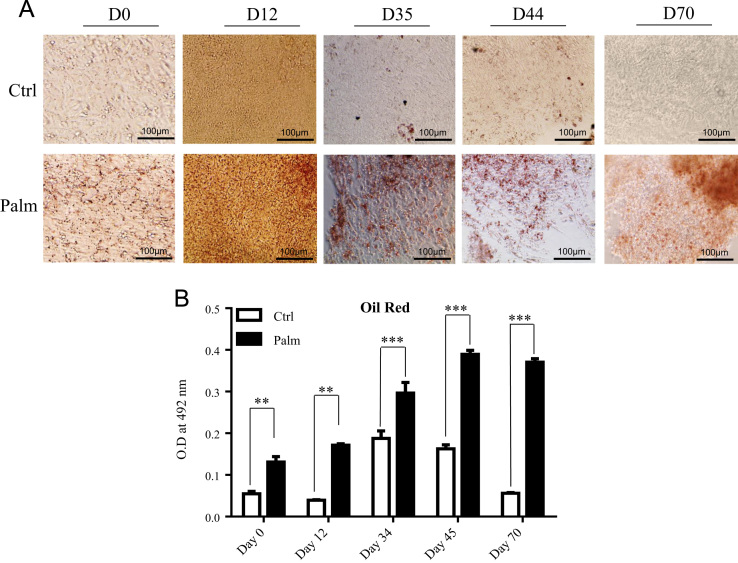
Quantitation of palmitate uptake by hESCs during different stages of *in vitro* embryonic neuorgenesis by Oil red O staining. (A) The H9 cells were differentiated into cortical neurons using established protocol in the presence of palmitate (250uM) and vehicle control. The cells were fixed at indicated time points for Oil red O staining. The phase-contrast images (20×) were captured on EVOS XL Core microscope (scale bar = 100 μm). (B) The Oil red stain from cells, described in panel A was extracted and quantitated using standard protocol and measured at absorbance 490 nm using TECAN Infinite M200 pro plate reader. The data (bars) are represented as mean ± standard deviation. * *p* ≤ 0.05, **p ≤ 0.01, ***p ≤ 0.001 (unpaired Student׳s *t* test).

**Fig. 3:**
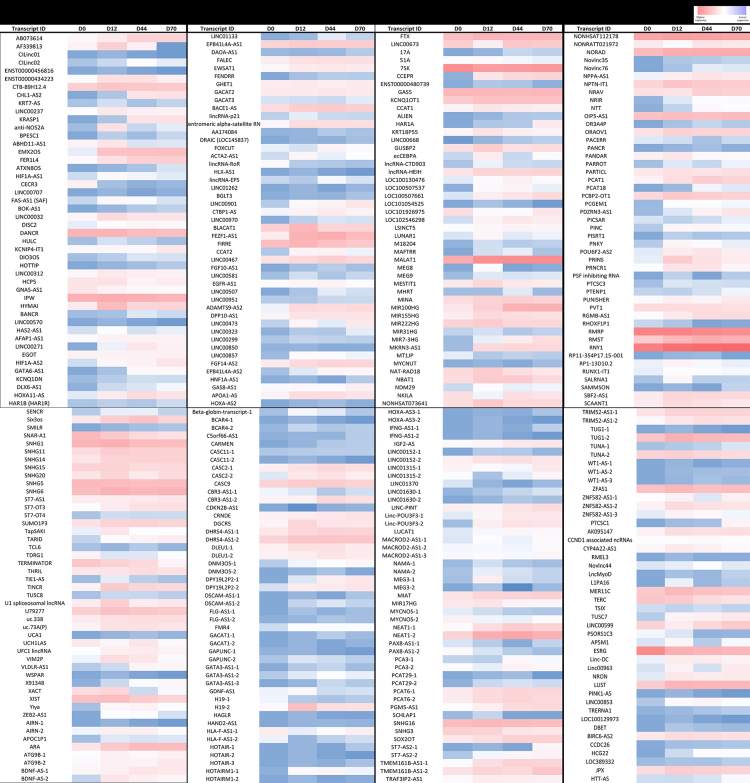
Expression profile of lncRNAs from different stages of *in vitro* embryonic neuorgenesis. The expression of 372 functionally known lncRNAs was analyzed by RT-qPCR using ‘nrStar™ Human Functional LncRNA PCR Array’ on H9 cells at days 0, 12, 44 and 70 of neural differentiation. The delta CT values for individual lncRNAs are shown after normalization to 18S rRNA. The bright red color indicates highest expression whereas bright blue shows lowest expression.

In this dataset, human embryonic stem cells (hESCs) were used as a model of embryonic neurogenesis. By their directed differentiation into neurons using established protocols [1], [2], [3], the expression profiles of select lncRNAs were assessed during *in vitro* embryonic neurogenesis (Fig. 3) and dataset was generated for differentially expressed lncRNAs due to excess fat uptake (Supplementary Table 1). The undifferentiated hESCs were treated with 250 µM palmitate after identifying it as the highest concentration which is non-toxic to these cells (Fig. 1) as this could lead to maximum effect of fat uptake without affecting cell viability. The palmitate treated hESCs were differentiated towards neurons in constant levels of palmitate throughout and fat uptake was confirmed by Oil Red O staining (Fig. 2A and B). The expression analysis of lncRNAs was performed by RT-qPCR on vehicle control and palmitate treated cells from 4 stages of differentiation, D0 (undifferentiated hESCs), D12 (neural stem cells), D44 (neural progenitors) and D70 (neurons) using lncRNAs array plates from Arraystar Inc. which contains 372 functionally identified lncRNAs found to be associated with lipid metabolism and other pathways (Cat# AS-NR-004). Fig. 3 shows data on expression profile of these lncRNAs whereas Supplementary Table 1 contains list of these lncRNAs differentially expressed in the presence of palmitate (250 µM) relative to vehicle control at D0, D12, D44 and D70 of neural differentiation.

## 2 Experimental design, materials and methods

### 2.1 Cell culture and fat uptake

The undifferentiated hESCs (H9 cells) were cultured in feeder free condition on Matrigel coated 24-well tissue culture plates (Corning, Cat. # 3527) in mTesR1 media with palmitate treatment as described [1], [4].

### 2.2 Expression analysis of lncRNAs

The expression analysis of lncRNAs was performed on palmitate treated and vehicle control cells from four stages of neural differentiation of hESCs, D0, D12, D44 and D70 using Long non-coding RNAs (lncRNAs) array plates were purchased from Arraystar, Inc. - USA (Cat# AS-NR-004) as described [1].

### 2.3 Oil Red O staining

To detect lipid uptake by cells at different time points of neural differentiation, 4-well plates containing cells treated with palmitate or vehicle control at D0 to D70 were stained with Oil Red O (Sigma, Cat# O0625) and quantified as published [1], [5], [6].
