# Supplementary material for: Saturated fatty acid regulated lncRNA dataset during in vitro human embryonic neurogenesis
Source: Data Brief. 2018 Oct 27;21:1061–5. doi: 10.1016/j.dib.2018.10.101 (PMC6226592; doi:10.1016/j.dib.2018.10.101)
Supplement: Supplementary file 1 — Supplementary material. [file mmc1.docx]

**Conflict of Interest**

This is to confirm that the materials submitted in the manuscript entitled ‘Saturated fatty acid regulated lncRNA dataset during *in vitro* human embryonic neurogenesis’ are original and authors, Mustafa T. Ardah, Shama Parween, Divya S. Varghese, Bright Starling Emerald, and Suraiya A. Ansari of this manuscript are in agreement to have the article published in this journal. All of the authors declare that they have no conflict of interest.

Sincerely,

Suraiya Ansari
